# Development of Novel Platform to Predict the Mechanical Damage of a Miniature Mobile Haptic Actuator

**DOI:** 10.3390/mi8050156

**Published:** 2017-05-13

**Authors:** Byungjoo Choi, Jiwoon Kwon, Yongho Jeon, Moon Gu Lee

**Affiliations:** 1 Department of Mechanical Engineering, Ajou University, Suwon-si 16499, Korea; dasom@ajou.ac.kr (B.C.); princaps@ajou.ac.kr (Y.J.); 2 Department of Convergence Technology Research, Korea Construction Equipment Technology Institute, Gunsan-si 54004, Korea; jwhj0814@gmail.com

**Keywords:** drop test, impact analysis, reliability, haptic actuator, linear resonant actuator (LRA)

## Abstract

Impact characterization of a linear resonant actuator (LRA) is studied experimentally by a newly-developed drop tester, which can control various experimental uncertainties, such as rotational moment, air resistance, secondary impact, and so on. The feasibility of this test apparatus was verified by a comparison with a free fall test. By utilizing a high-speed camera and measuring the vibrational displacement of the spring material, the impact behavior was captured and the damping ratio of the system was defined. Based on the above processes, a finite element model was established and the experimental and analytical results were successfully correlated. Finally, the damage of the system from impact loading can be expected by the developed model and, as a result, this research can improve the impact reliability of the LRA.

## 1. Introduction

Haptic perception can be classified into kinesthesia and tactility. The kinesthesia senses the mass, hardness, and shape of an object, whereas tactility senses the roughness, protuberance, and temperature of an object surface [1]. The haptic actuators which can communicate between a human and a machine based on the sense of touch are being developed. Several studies have recently focused on how to make this technology more realistic and immersive. Therefore, its field of application is expected to expand further. 

Many studies have carried out the technology related to haptic perception over recent decades. The use of an eccentric rotating motor (ERM) as a vibrational source is reduced because of a lack of sensuous delivery caused by its slow response and narrow frequency range [2,3]. The solenoid resonant actuator (SRA) and piezoelectric resonant actuator (PRA) are introduced as having fast and wide frequency responses. However, the SRA is 25 mm or longer, making it too bulky to be applied to a small device, and the PRA requires a high-voltage amplifier and lacks structural durability because of the occurrence of the piezoelectric effect at high voltages and their embrittlement. In recent years, consequently, the LRA, with good durability and reasonable operating voltage, has been used by employing a mechanical spring. It can achieve the fast response and wide vibrational frequency range by using a small-sized voice coil motor (VCM) [4]. Generally, haptic actuators are constantly applied to mobile devices, medical instruments, automobiles, and entertainment devices. Additionally, high-definition (HD) haptic actuators are being developed for them. However, there are various impacts in the area where haptic actuators are applied. Due to these impacts, research is needed to make this device reliable. Such efforts have been made in related industries, but there is a need to make systematic forecasts because the existing ones are based on the trial and error method with the designer’s experience.

**Figure 1:**
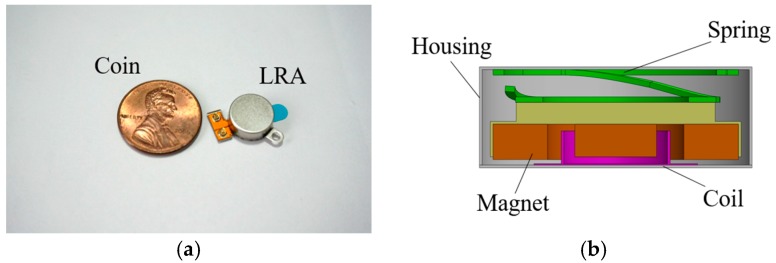
Appearance and internal structure of a coin-type LRA: (**a**) appearance; and (**b**) cross-sectional view.

Figure 1 shows the structure of an LRA (iPhone 4s, vibration motor). A spring, magnet, and moving mass are fixed on the top of the housing, while the coil is attached at the bottom of the housing. When a current is applied to the coil, a Lorentz force is generated due to the electromagnetic interaction between the current of the coil and the magnetic field. This force results in haptic perception by the vibration of the magnet and moving mass attached to the spring [5]. The structural durability of each component is a crucial part in the delivery of consistent haptic perception and, therefore, the related industry requires high reliability for this system. In the case of the impact durability test dropping from 1.8 m, from a human ear’s height, only a 10% malfunction rate for the haptic actuator mounted in a smartphone is allowed in the industry. In order to satisfy this criterion, many suppliers have performed several research activities, such as reliability testing and analytical work [6,7,8,9,10,11,12]. The drop test, which is one of the experimental methods, is the most accurate way to study the impact resistance of a mobile device. However, experimental approaches cannot effectively characterize the impact behavior of each component because of its small size and behavior under a high-rate regime. In order to solve this issue, the finite element analysis was introduced in this study and this approach was verified by a comparison with the experimental approach.

Among the components in the actuator, the spring is the most sensitive part under impact loading and the impact damage can change the natural frequency of the spring, causing a malfunction, like insufficient acceleration.

In this study, we developed the novel platform which can predict the mechanical damage for LRA under impact loading. To generate the impact loading, the specific drop tester was developed and the repeatability test was executed for checking the feasibility of the test apparatus. Additionally, the analytical model was established and appropriate material testing was performed for obtaining the mechanical property simultaneously. As the relevance of the analytical model was proved by a correlation with experimental results, we can finally successfully predict the mechanical damage of the LRA under impact loading with the developed analytical model. We can assure that the research output explained in this article can play a significant role for damage analysis of various electrical devices under impact loading.

## 2. Drop Tester 

### 2.1. Drop Tester Configuration

In general, the falling motion of the mobile device shows several aspects by rotation moment, air resistance, secondary impact, and so on. Therefore, it is very difficult to analyze the mechanical behavior experimentally due to the experimental uncertainty caused by the falling motion. In order to control the experimental uncertainty, a new drop tester was developed.

**Figure 2:**
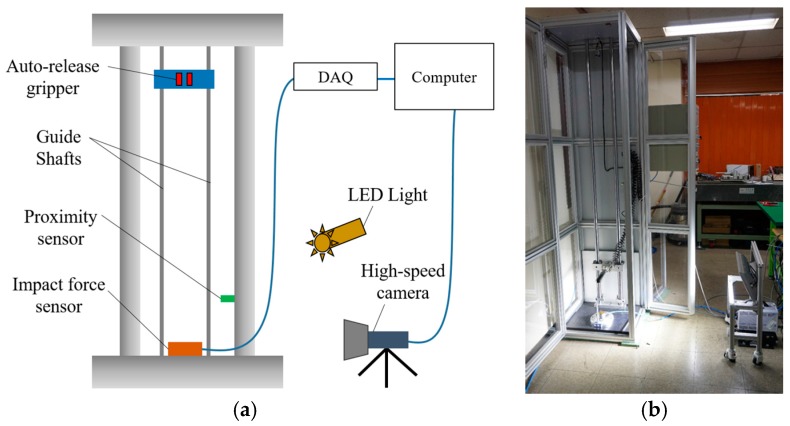
Drop tester configuration: (**a**) schematic diagram; and (**b**) experimental setup.

The experimental setup was constructed as shown in Figure 2. The impact force sensor (200C50, PCB Piezotronics Inc., Depew, NY, USA) can measure the frequency band of 0.0003 Hz to 30 kHz and a maximum dynamic compression force of 222.4 kN [13]. It was mounted onto the bottom of the test system. The measured signal was acquired and saved by a data acquisition system (LABVIEW, National Instruments, Austin, TX, USA). Additionally, the high-speed camera (FASCAM, APX-RS, Photron, Tokyo, Japan) was introduced for capturing the impact behavior visually and the image was captured at 512 × 512 resolution and 10,000 fps with an LED light.

The test specimen was fixed with an auto-release gripper, which was operated by a pneumatic system, and the gripper was attached to the bushing. When the test begins, the bushing starts falling down through the guide shaft and then the gripper releases the specimen passing the proximity sensor. Finally, the test object impacts the force sensor mounted onto the bottom part of the impact tester. To reduce the experimental uncertainty, especially friction during the falling of the object, the bushing was composed of monomer cast nylon and grease was applied onto the guide shaft.

### 2.2. Drop Tester Verification

The developed drop tester consists of several supplementary components for controlling the experimental uncertainty and this supplementary component can distort the impact behavior. Therefore, the feasibility of this test apparatus must be verified in an appropriate manner. In this study, we fulfilled the verification of this feasibility by a comparison with the free fall test. A slender rod was used as a specimen and this rod was wrapped with silicone rubber to mitigate the vibration. Both the rod with and without the constraint were dropped from a height of 1.8 m. This height is a simulated value of the height of human ears. The drop from this height will result in a severe impact on the devices.

**Figure 3:**
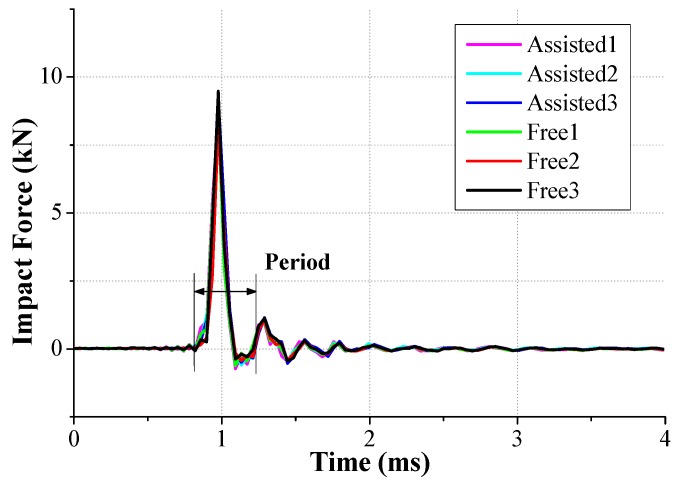
Comparison of the generated impact force between the free and assisted tests.

Despite the small allowable error in the peak force and period, as shown in Figure 3 and Table 1, the primary impact and subsequent oscillation for both tests was well matched and the agreement between the free and assisted tests suggests that the test results with the newly-developed test apparatus shows good repeatability.

**Figure 4:**
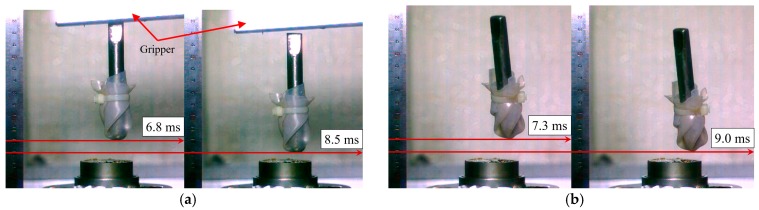
Comparison of falling velocity from the free and assisted tests: (**a**) assisted fall; and (**b**) free fall.

For the comparison of the falling velocity just before the impact, the traveling time for a distance of 10 mm was measured with a high-speed camera, as shown in Figure 4. The traveling time was estimated as 1.7 ms for both tests. Therefore, we can conclude that the falling velocity is 5.88 m/s and this velocity can be used as the initial velocity for finite element analysis.

## 3. Finite Element Analysis of Impact Behavior of the LRA

### 3.1. Determination of Damping Ratio for the Spring

The damping ratio of the mechanical components is a major factor for determining the magnitude of the structural response under external loads. However, it is difficult to develop the constitutive model for structural damping because this is fully reliant on the dynamic condition. In this study, we used the experimental approach rather than an analytical approach to obtain the damping ratio of the spring. We applied a random excitation signal to the spring and extracted the vibrational peak ($x_{i}$) and peak ($x_{i+1}$) at a certain time. The logarithmic decrement method is helpful to obtain the damping ratio by applying the extracted peaks to Equations (1) and (2) [14]:

(1) $$\delta =\ln\frac{x_{1}}{x_{2}}$$

(2) $$\xi =\frac{\delta }{\sqrt{{\left(2\pi \right)}^{2}-\delta^{2}}}$$

**Figure 5:**
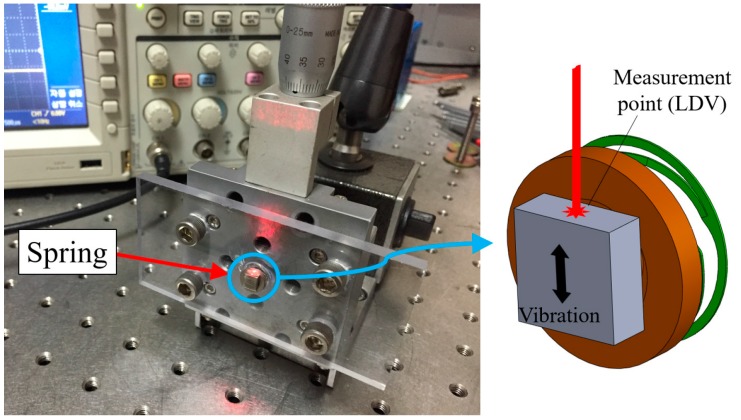
Experimental setup for measuring the vibrational behavior of the spring.

**Figure 6:**
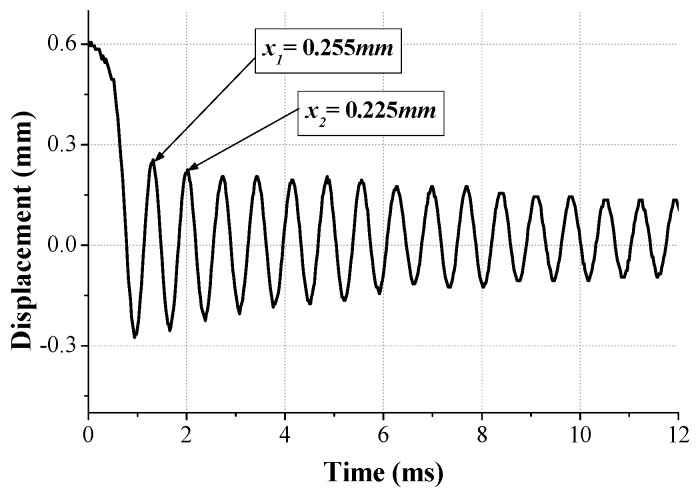
Measuring data for vibrational behavior.

Figure 5 shows the experimental setup for measuring the vibrational behavior of the spring with a moving mass and the data was obtained by laser Doppler velocimetry (LDV). The direction of the vibration and excitation was matched with the falling direction of the dummy phone. Since the moving mass of the LRA is very small and the reflection characteristic is low, it is difficult to measure with laser. Then, the hexahedral magnet was attached to the upper part of the moving mass, so that the laser measurement focus was positioned on the side. The measurement was run on a vibration isolation stage (vibration isolation system, DAEIL SYSTEMS) to mitigate any vibrational noise and the data was acquired by an oscilloscope (Tektronix, Beaverton, OR, USA). As shown in Figure 6, the magnitude of the first peak was 0.255 and the second one was 0.225; therefore, the damping ratio (ξ) of the spring was eventually calculated as 0.02 using the logarithmic decrement method. 

### 3.2. Micro-Tensile Test for Spring

**Figure 7:**
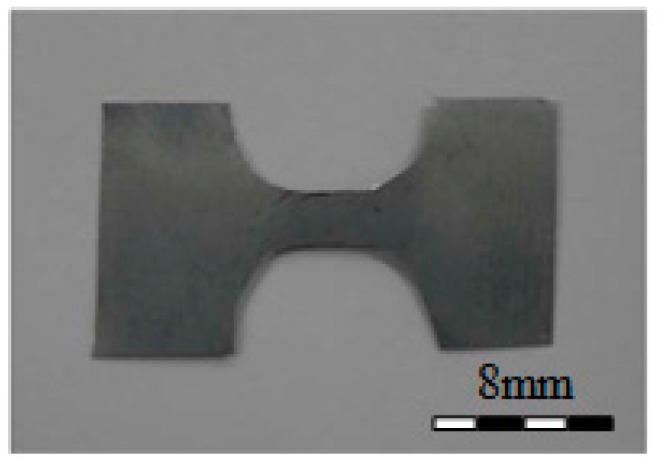
LRA spring test specimen.

**Figure 8:**
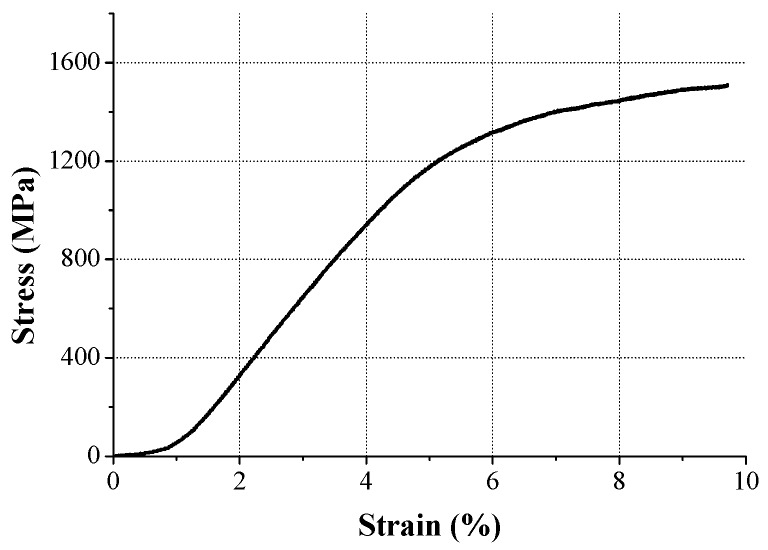
Stress–strain curve of SUS301 obtained by the micro-tensile test.

The LRA spring in this study is a thin plate 100 μm thick and 9 mm in diameter. The mechanical property of the thin plate is quite different from a bulk material because this can be changed by the correlation of the grain size with the component size [15]. Therefore, the micro-tensile test with a specially-prepared specimen was conducted to understand the mechanical property of the spring. The material of the specimen was SUS301 and this specimen was prepared with 200 μm thick, 3 mm gage length, and 19 mm total length using photo etching as shown in Figure 7. A universal material testing machine (UT-005, MTDI, Dajeon, Korea) was used in the micro-tensile test and the stress–strain curve of the SUS301 is shown in Figure 8. Even though the ultimate tensile strength (UTS) of SUS301 is known to be 1300 MPa [16], the UTS in this micro-tensile test was measured as 1510 MPa because of the size effect.

### 3.3. Experimental Verification of Analytical Model

**Figure 9:**
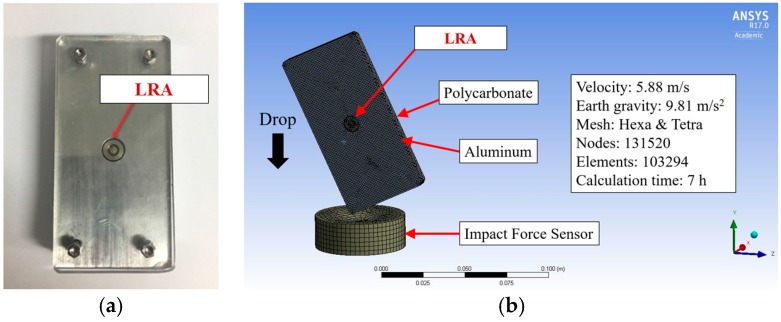
Physical and FE model of dummy phone with LRA: (**a**) dummy phone as a test specimen; and (**b**) finite element analysis.

To mimic the cellular phone, the appropriate dummy phone was prepared and the finite element (FE) model was also generated for this physical model, as shown in Figure 9. The dummy phone was composed of an aluminum panel and the LRA was attached to the center of the panel. In general, the LRA is covered with a metal housing. This makes it difficult to measure the vibration of the moving mass. Therefore, the LRA housing was removed to observe the moving mass movement during the impact moment. Additionally, this aluminum panel was covered by a transparent plate made of polycarbonate. This transparency allows the capturing of the behavior of the LRA by a high-speed camera. The hexa and tetra element was applied as the FE model element and a fine mesh with a 60 μm element size was applied in the spring and moving mass, which was our main interest. The impact that a haptic actuator dropped from the 1.8 m ear’s height to the floor is a significant problem. In this case, a falling velocity was applied as 5.88 m/s, which was measured from the drop test just before collision, and gravitational acceleration was defined as 9.81 m/s^2^. Futhermore, the friction coefficient was applied as 0.61 for static and 0.47 for kinetic, repectively.

**Figure 10:**
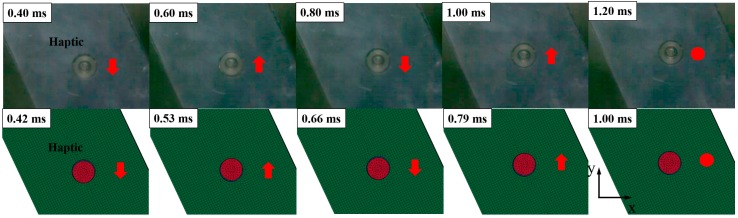
Impact behavior of the LRA in the drop test and FE simulations.

We can identify the rebounding behavior of the moving mass from the high-speed camera and FE simulations, as shown in Figure 10. The movement was basically a relative motion of the dummy phone. The motion was measured by analyzing the high-speed image with the post-processing software Image J. A dot tracking method was applied to the center of the moving mass in the test. After numerical computation, tracking data was compared with the displacement of the center mesh from the finite element analysis (FEA) results. The moving mass was oscillated with 0.40 ms period in the drop test whereas the oscillating period was 0.24 ms in the FE simulation. Despite this discrepancy of the oscillating period, we can insist that the FE model was valid for this application because the moving mass in both cases was stabilized after two periods and the rate dependency of the metallic structure can be negligible.

**Figure 11:**
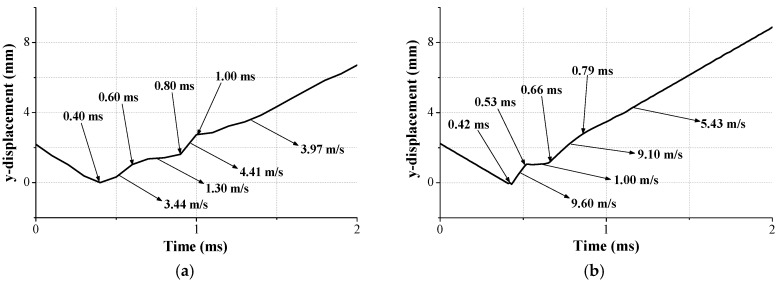
Comparison of traveling distance and moving velocity of the spring in the LRA: (**a**) drop test; and (**b**) FE simulation.

To clearly understand the validity of the FE simulation, the traveling distance of the moving mass is presented in Figure 11. We can confirm that the trend of both cases was well matched even though there was no energy dissipation in the FE simulation caused by perfectly elastic modeling.

**Figure 12:**
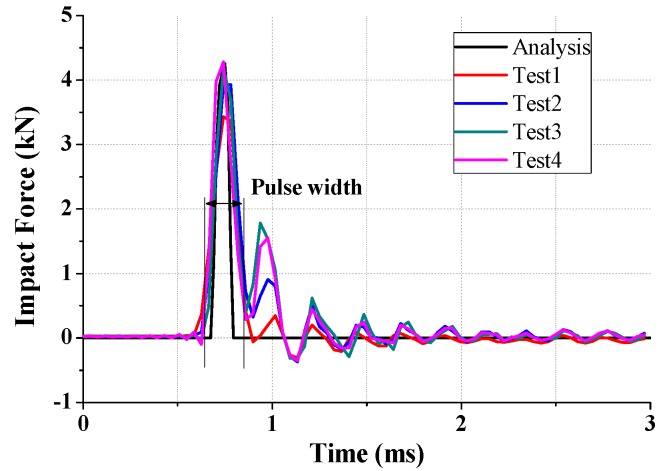
Impact force from the drop test and the FE simulation.

In spite of the short period of time, the estimation of the accuracy of the impact force is very important because it can cause external and internal damage to the structure. Therefore, the agreement of the impact force from the drop test and the FE simulation must be checked. The peak force from the drop test and the FE simulation was measured and verified the validity of the analytical model as shown in Figure 12 and Table 2. The four tests were conducted and the average peak force was measured as 3959.78 N with a 329.64 standard deviation. This value corresponds to the FE simulation results. On the other hand, it has the small error (7.5%) from drag and friction forces.

### 3.4. FE Simulation

**Figure 13:**
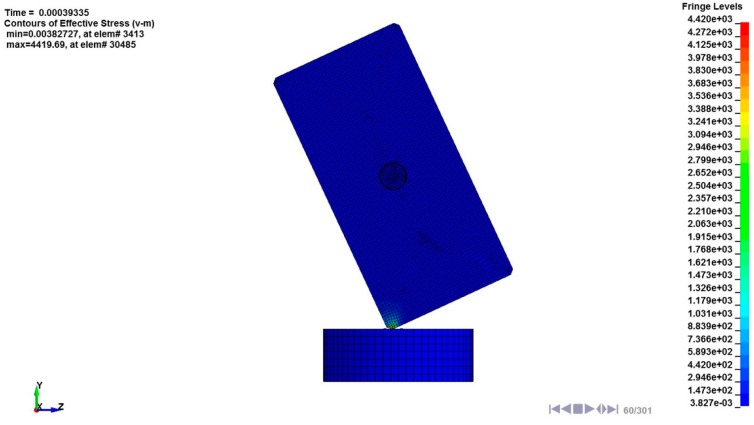
Stress (von Mises) contour from the FE simulation under impact loading.

When a smart device, such as smartphone, drops on the floor, it starts the rotation due to rotational momentum generated by its asymmetric mass distribution. This rotation can increase the uncertainty and make appropriate analysis difficult. Therefore, the rotational motion of the dummy phone used in this study was restrained to avoid this uncertainty and to obtain the appropriate simulation results during the FE simulation. In the FE modeling, the vertical line on the ground was considered as the datum line, and the mass distribution of the dummy phone, which was the impacted object, was bilaterally symmetrized with respect to this datum line. At the rebounding state, we can achieve the translational motion of the impacted object without any rotational motion using this FE model. The stress contour from the FE simulation is shown in Figure 13. The impact occurred at 0.33 ms and maximum stress was observed as 4420 MPa at 0.39 ms. The stress was concentrated in the vicinity of the impact point and this concentration phenomenon was verified as observing the damage in the experiment. At 0.46 ms, the impacted object began rebounding and the LRA, including the moving mass and spring components, started the oscillation. The traveling behavior of the LRA spring attached to the impacted object is already shown in Figure 11b.

**Figure 14:**
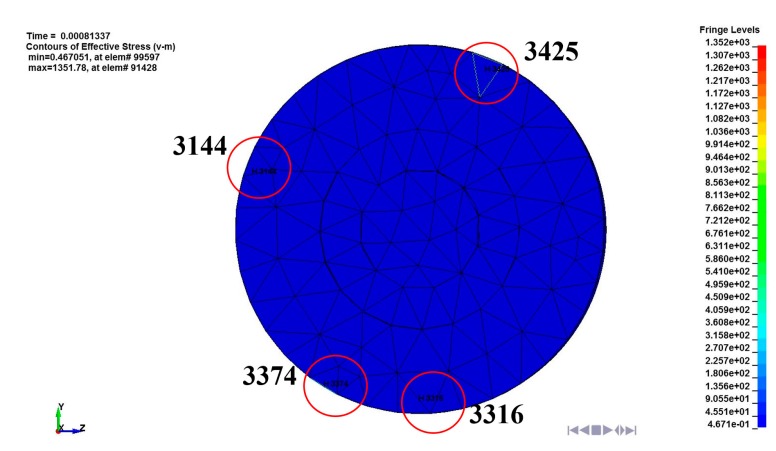
Contact element of the moving mass fixed to the spring in the LRA.

**Figure 15:**
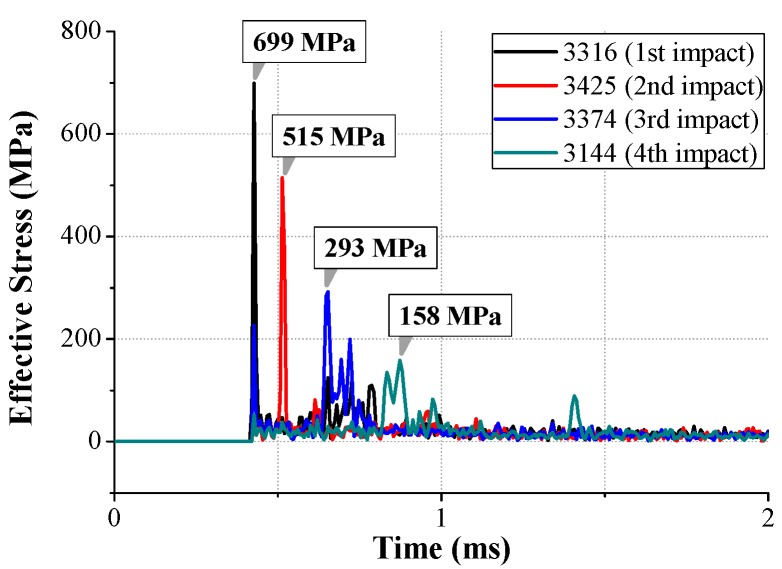
Impact stress (von Mises) generated in contact elements of the moving mass (from Figure 14).

In general, any component attached to a traveling object subjected to perpendicular motion is expected to travel along the same direction with the main object. In other words, the motion of the LRA can be expected to be in perpendicular motion to the ground in this study. In the FE simulation, however, the moving mass had asymmetrically oscillated to the datum line because of the asymmetricity of the spiral spring fixed to the moving mass. Therefore, the impact between the moving mass and the LRA housing was also asymmetric. Even though the first impact occurred perpendicular to the ground between element 3316 and the housing, all of the impact after the first one appeared as a rotational motion in the clockwise direction as shown in Figure 14. The impact stress of each element is shown in Figure 15 and the maximum stress was 699 MPa (0.43 ms), 515 MPa (0.51 ms), 293 MPa (0.65 ms), and 158 MPa (0.87 ms), respectively. This stress may cause spring damage.

**Figure 16:**
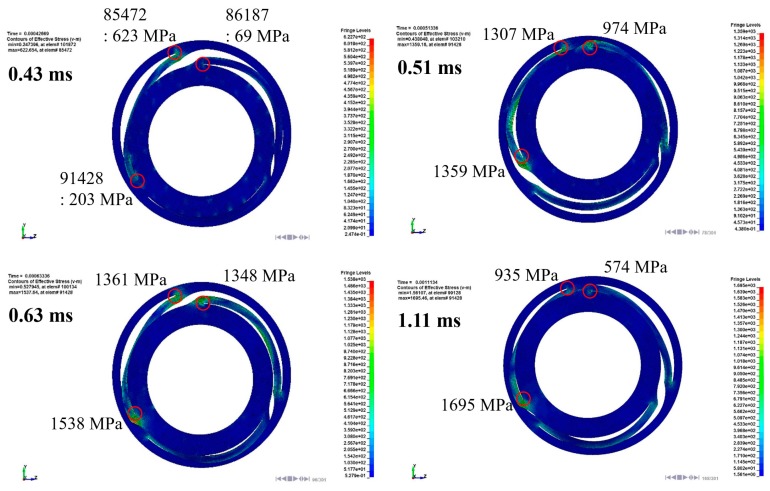
Impact stress (von Mises) and deformation of the spring in the LRA during impact loading.

In order to understand the spring behavior, the mechanical behavior of the spring was investigated. The stress and deformation state at a certain time is described in Figure 16. As mentioned above, the structural asymmetricity makes the vibration skew to the left. Due to the spring geometry, the stress was concentrated to the leg, which is in the vicinity of the supporting point, and the maximum stress was observed at elements 85,472, 86,187, and 91,428, corresponding to each leg. The downward maximum deformation of the spring occurred at 0.43 ms. At that instant, elements 85,472 and 86,187 were subjected to the tensile stress and the maximum stress was calculated as 623 MPa at element 85,472, whereas element 91,428 was subjected to the compressional stress and the stress value was observed as 203 MPa. On the other hand, the upward maximum deformation of the spring occurred at 0.51 ms. At that instant, the stress state of each element was reversed and the stress value was magnified by stress accumulation. The stress values of elements 85,472 and 86,187 were 1307 MPa and 974 MPa, respectively, and the stress value of element 91,428 was 1359 MPa. During the whole transient state, the maximum stress occurred in the upward bouncing of the second period. The measured value was 1695 MPa, which is over the UTS of the spring and, therefore, we can expect severe damage to the spring [17].

**Figure 17:**
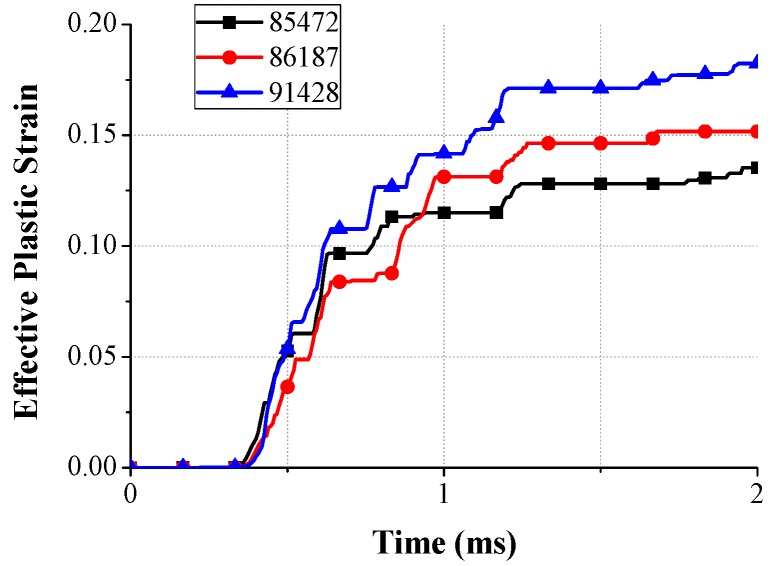
Effective plastic strain for certain elements in the spring during impact loading.

Figure 17 illustrates the effective plastic strain for certain elements subjected to concentrated stress. The plastic deformation began at 0.35 ms as the impact was transmitted to the spring. The initial strain slope of each element was similar, but the strain was increased due to the high stress condition after the first period (0.63 ms). Even the strain of element 86,187 overtook the strain of element 85,472 at 1.0 ms because the accumulation and dissipation of the impact energy was different for each element during the transient state. The maximum strain was calculated as 0.135, 0.182, and 0.152 at element 85,472, 91,428, and 86,187, respectively.

**Figure 18:**
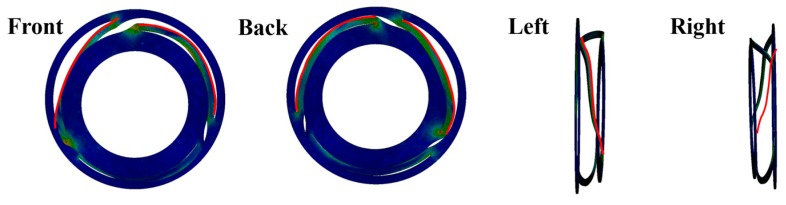
Expected deformation shape (highlighted with red lines) of the spring after the impact.

The expected deformation shape of the spring was calculated by FE simulation and this is illustrated in Figure 18. The upper plate, which is the spring leg, is severely deformed and, therefore, we can easily expect that the vibrational characteristic of the LRA is changed. This means that this LRA is no longer providing the haptic perception.

## 4. Conclusions

We developed a novel platform which can predict the mechanical damage for an LRA under impact loading by using drop test and FE analysis methods.

(1) For the analysis, a series of preparations were carried out. First, the drop tester was newly-developed for experimental verification of the FE model. Its experimental verification satisfied the free fall conditions while assisting the drop with a test apparatus. Second, a micro-tensile test was performed to obtain the material properties considering the size effect of the thin LRA springs. Third, structural damping was modeled by measuring the vibration displacement of a spring with the excitation signal.
(2) Based on the previous study, the impact FE modeling of a dummy phone including an LRA was performed, and its experimental verification was carried out by comparison of the impact deformation and force during the impact behavior. Despite the error in the impact force (7.5%) and pulse width (33%), the analytical model and experimental model were well correlated. Additionally, the impact rebound displacement is well matched.
(3) Consequently, the damage of the FE model was analyzed. The external impact and secondary internal impact of the LRA moving mass were concentrated on the LRA spring. Primary and secondary impact generated a maximum impact stress of 1695 MPa. Further, effective strain at the same position was evaluated as 0.182. The damaged shape of the spring was confirmed and a vibration characteristic change was expected.

In conclusion, impact deformation and force were calculated through an experimentally-verified FE model. This process can redeem the durability study against impact which has been conducted by the designer’s experience and trial-and-error. Finally, this research will be used extensively in impact analysis of smart devices, automobiles, medical instruments, game machines, and remote controls, including miniature parts.

## Figures and Tables

**Table 1:** The peak impact force and period from the free and assisted tests.

No.	Peak Impact Force (kN)	Period (ms)	Force Error (%)
Assisted1	8.68	0.39	2.36
Assisted2	8.84	0.47	0.56
Assisted3	9.23	0.47	3.82
Free1	8.78	0.39	1.24
Free2	8.31	0.43	6.52
Free3	9.48	0.43	6.64

**Table 2:** Comparison of the impact force from the drop test and the FE simulation.

No.	Peak Force (N)	Pulse Width (ms)
Analysis	4255.94	0.083
Test1	3433.23	0.128
Test2	3934.92	0.126
Test3	4186.65	0.118
Test4	4284.30	0.121
